# Myoma Expulsion after Uterine Artery Embolization

**DOI:** 10.1155/2021/6644229

**Published:** 2021-09-08

**Authors:** Vivian Fernanda do Amaral, Fernanda Yumi Yochiy, Mário Luiz Furlanetto, Spencer Luiz Marques Payão

**Affiliations:** ^1^Department of Medicine of Faculty of Medicine of Marilia, 17519-060, Marilia, SP, Brazil; ^2^Department of Vascular and Neurovascular Interventional Radiology (SIRVAN), 17515-001 Marília, SP, Brazil

## Abstract

**Introduction:**

Uterine leiomyomas are the most common benign pelvic tumors in women over 35 years and can be symptomatic or asymptomatic. Among the main treatment strategies, there are hormone therapy, hysterectomy, myomectomy, and uterine artery embolization (UAE), a recent and promising treatment for patients who wish to avoid hysterectomy. Ideal candidates for UAE are women with symptomatic uterine leiomyomas that present no desire for pregnancy, premenopausal and heavy menstrual bleeding, or dysmenorrhea caused by intramural fibroids. *Case Presentation*. A 36-year-old female diagnosed with leiomyomas and an extensive history of failed previous treatments who, in order to preserve her uterus, underwent UAE and had tumor expulsion 15 days after the procedure. The patient remained eight months in amenorrhea and, currently, presents normal hormone levels and irregular periods.

**Conclusion:**

UAE presents itself as a minimally invasive procedure and as an efficient alternative for those patients who wish to preserve their uteri and also improve their symptoms and quality of life.

## 1. Introduction

Uterine leiomyomas are the most common benign pelvic tumors in women over 35 years and are more commonly found among African-descendant females [1, 2]. They are fibromuscular growths of the myometrium with an incidence that varies from 20% to 25% in women of reproductive age and can be symptomatic or asymptomatic [3–5].

Among the main treatment strategies, there are hormone therapy, hysterectomy, myomectomy, and uterine artery embolization (UAE) [6]. The lack of randomized controlled trials is one of the factors that makes the creation of guidelines for treatment very difficult. The use of embolization of uterine artery to reduce uterine fibroid volume was first observed in 1995, and since then, it has become a promising treatment option and minimally invasive, for patients who wish to preserve their uterus [7–10].

When selecting or choosing UAE, the symptoms presented and the tumors' size, number, and location must be taken into consideration. Ideal candidates for UAE are women with symptomatic uterine leiomyomas that present no desire for pregnancy, premenopausal and heavy menstrual bleeding, or dysmenorrhea caused by intramural fibroids. Despite that, it is feasible that the decrease of fibroid in size, even without expulsion, can restore uterine characteristics, favoring a future pregnancy. The UAE is absolutely contraindicated in cases of asymptomatic fibroids, uterine malignancy, pregnancy, and pelvic inflammatory disease. Just like any other procedure, complications can happen such as fever, pelvic pain, vaginal discharge, uterine and/or ovarian insufficiency, and mass expulsion. We present a case in which the patient's tumor expulsion occurred 15 days after UAE [11].

## 2. Case Presentation

A 36-year-old afrodescendant female was referred to us with metrorrhagia, menorrhagia, dysmenorrhea, and dyspareunia of ten-year duration. The patient also reported weakness, fatigue, and secondary anemia. She was diagnosed with uterine fibroids, and during that period of time, she was submitted to numerous hormone therapies and four unsuccessful myomectomy procedures. Notwithstanding, she underwent over 20 blood transfusions over that 10-year period.

MRI (Magnetic Resonance Imaging) performed a year prior to UAE demonstrated an enlarged, myomatous uterus with a volume of approximately 330 cm^3^ and the presence of numerous sparse myomatous nodules. The main ones are a posterior intramural fibroid on the right measuring 21 × 14 mm and a submucous fibroid, measuring 65 × 54 × 56 mm, filling the uterine cavity in its totality and the upper third of the cervix.

A preoperative transvaginal ultrasound (US) revealed a 607.40 cm^3^ uterus, the presence of sparse myomatous nodules, the biggest being a submucous fibroid, measuring 67 × 60 × 67 mm (141 cm^3^), and a posterior subserous fibroid, measuring 38 × 20 mm.

The patient was referred to us by her gynecologist and given the option to pursue either hormonal therapy, uterine fibroid embolization, or hysterectomy. She opted for UAE, due to uterine preservations and further benefits.

**Figure 1:**
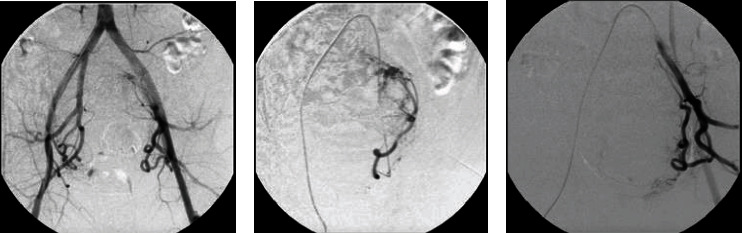
(a) Periprocedural digital subtraction angiography from UAE, showing aorta-iliac bifurcation and left (b) and right (c) uterine arteries before embolization. The procedure was performed on the left with four vials of Embospheres, two of 300-500 microns, one of 500-700 microns, and one of 700-900 microns. On the right, three vials were used, one of 300-500 microns, one of 500-700 microns, and one of 700-900 microns.

**Figure 2:**
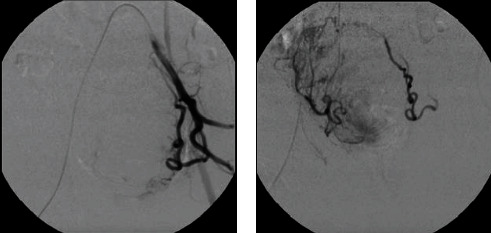
Digital subtraction angiography postembolization demonstrating effective embolization of the left (a) and right (b) uterine arteries.

A periprocedural arteriography revealed pervious distal aorta, common, internal, and external iliac arteries, and a giant uterine fibroid with its blood supply coming from both uterine arteries (Figure 1). A standard UAE was performed, and the uterine arteries were embolized with 300 to 900 microns until they were completely devascularized (Figure 2). Postprocedural arteriography demonstrated endpoint sign, proving technique and therapeutic successes. The patient remained in postoperative care for 2 days, and analgesics were prescribed at discharge.

**Figure 3:**
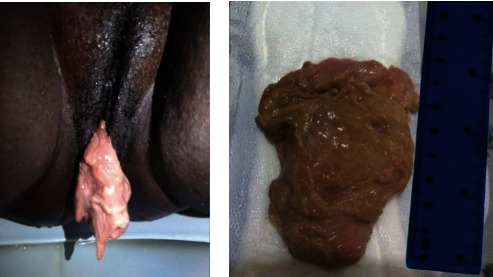
(a) Vaginal expulsion of infarcted submucous fibroid 15 days after uterine artery embolization. (b) Specimen measuring approximately 8 cm.

Seven days after discharge, the patient presented with abdominal contractions, low back pain, cervix dilation, and the beginning of tumor exteriorization, simulating labor. The complete mass expulsion occurred, at the patient's home, eight days after symptom onset and measured approximately 8 cm (Figure 3). The patient underwent dilation and curettage to remove residual myoma along with suppurative exudate. The patient was discharged on the same day with antibiotics and analgesics. Transvaginal US five months after UAE demonstrated uterine volume reduction to 99.8 cm^3^, adenomyosis signs, and hypoechoic and hyperechoic intramural posterior nodules measuring 25 × 14 mm and 18 × 14 mm, respectively.

The patient is currently asymptomatic; reports quality of life, self-esteem, and sex life improvements; remained in amenorrhea for eight months after the procedure, with hormone levels within the normal parameters; and currently presents herself with irregular periods.

## 3. Discussion

Uterine fibroid diagnosis by anamnesis, physical examination and imaging exams, selection of patients who meet the criteria for UAE, and patient referral to Vascular Surgery or Interventional Radiology Departments must be done by gynecologists. The imaging exams, such as US and MRI, are essential to collect more precise information concerning the tumor's size and location but also to rule out other diseases that mimic fibroids' symptomatology, for example, endometriosis and adenomyosis. The volume of both uterus and leiomyomas must be taken into consideration when indicating UAE; however, there is not a well-determined maximum volume limit, since patients with bulky uteruses can benefit from the procedure clinically. Leiomyomas can be classified based upon their location within the uterine wall into four types: submucous, intramural, subserous, and transmural. Pedunculated submucous, transmural, and submucous tumors have a higher risk for expulsion after embolization; therefore, determining the precise location of the fibroid is extremely important [12–14].

Knowledge in anatomical variations of uterine artery's origin is extremely necessary, especially for surgeons and interventional radiologists. Anatomical alterations found during UAE can be responsible for endometrium blood supply shift, resulting in possible conceiving difficulties. This is due to implantation problems, consequence caused by vascular insufficiency, and extracellular matrix alterations and release of cytokines and vasoactive substances [15–17]. Generally, specific catheters are introduced unilaterally or bilaterally in the femoral artery until they reach the aortic bifurcation level. Posteriorly, the contralateral iliac artery is accessed in order to identify the uterine artery's origin [18].

Women can present a wide range of symptoms, including metrorrhagia, pelvic pressure, and pain due to mass growth and reproductive dysfunction, which interfere with quality of life and sex life [12, 13]. Randomized controlled trials that enrolled patients with equivalent age, race, body mass index (BMI), parity, pregnancy, symptoms, and duration of symptoms show insignificant differences between patients who underwent hysterectomy compared to those who underwent UAE. The UAE success rate was 88.9%, and the failure rate was 11.1%, 6.2% of which were caused by technical unilateral failure and the remaining 4.9% by bilateral failure. In addition to that, urinary tract infections and urinary retention seem to be more common in patients undergoing hysterectomy than UAE. Furthermore, studies have shown that UAE patients had significantly less blood loss compared to those who underwent hysterectomy and also the total length of hospital stay is relatively shorter in patients undergoing UAE, about two days, when compared to the hysterectomy group, with an average of five days [18].

Comparative economic assessment demonstrated UAE patient's overall cumulative cost is slightly lower than those undergoing hysterectomy. Furthermore, postsurgical satisfaction comparison among hysterectomy and uterine artery embolization showed no difference, meaning both groups were equally satisfied or dissatisfied with their procedures and outcomes. Common complications such as fever and pain can be found in both groups. The rates of so-called major complications, such as pulmonary embolism, are rare and were also observed in both groups; and minor, such as postpuncture hematomas, thrombus in the gluteal artery, and nausea, do not differ significantly between the two groups. Literature data, however, is limited, and therefore, it becomes infeasible to draw conclusions about the effectiveness of UAE compared to hysterectomy, the standard procedure nowadays to eliminate the most common uterine fibroid's symptoms [18, 19].

In the case presented, after four unsuccessful abdominal myomectomies, numerous blood transfusions, and unsuccessful hormone therapy attempts, the patient refused hysterectomy surgery treatment and was referred, by her gynecologist, to the Department of Angiology and Vascular Surgery, to undergo UAE. Research carried out by the American Journal of Obstetrics and Gynecology with 968 women (ages 29-59 years) pointed out the majority of them, under the age of 40, find it necessary to have treatment options that preserve the uterus. And 84% of these indicated the importance of minimally invasive surgical treatments [6].

As with any procedure, it is important to highlight that the patient can experience postembolization syndrome, which consists of low-grade fever, pain, fatigue, nausea and vomiting, and complications, such as vaginal discharge, uterine and ovarian failure, and mass expulsion [20–22]. Myoma ischemia can lead to size decrease and symptoms' improvement; however, there is a possibility of mass expulsion as observed in our study. The management of infection and mass expulsion must be individualized taking into consideration the clinical condition of each patient, and the prevention for possible septic shock should be considered [18]. In the case presented, the patient had complete expulsion of the tumor 15 days after UAE that simulated labor process. The existing literature data, regarding mass expulsion of post-UAE fibroids, vary from 5% to 15% and the time period, from days to years. Of all cases of postprocedure expulsion, 95% are symptomatic, 89% of those were expelled in “bulk” form, which consists of total expulsion of the tumor or large pieces, and 11% were in “sloughing,” which consists of “melting” of the tumor surface or dissolving the tumor over a period of time [13, 23]. Our patient was discharged 2 days after the procedure, however came back seven days later, due to abdominal pain and tumor exteriorization. Therefore, in order to maximize patient's safety, it is important to take into consideration the leiomyoma's size and location to determine hospital stay, once a longer postoperative care may be necessary to assess and treat those possible complications.

The patient's improvement in the presented clinical case and the absence of further surgical procedures justify the success of the UAE. Even though studies show a small uterine reduction and normality of FSH levels three months after the procedure, these gain significance in the sixth month, in most cases. In the case presented, an 84% reduction in uterine volume and restoration of FSH parameters occurred over a period of 5 months [24].

The numerous unsuccessful procedures, the local chronic inflammatory process, and the psychological stress generated by years of treatment may justify a future difficulty to conceive. Studies that evaluated UAE outcomes show favorable results to pregnancy, with ovarian function preservation, uterine volume, and fibroid diameter decrease that reached 35% and 22%, respectively, after sixth months [25]. The indication of UAE for patients wishing to become pregnant is individualized, once hysterectomy does not meet personal interests, and myomectomy in the presence of several and/or large fibroids shows high morbidity, since it can lead to alteration of the myometrial anatomy, uterine cavity, and uterine tube insertion deformities. Therefore, the UAE becomes a viable option.

Eight months after the procedure, the patient reports absence of metrorrhagia, menorrhagia, dysmenorrhea, and dyspareunia and an improvement in quality of life, self-esteem, and sexual performance. Clinical follow-up after UAE is of extreme importance so that technical success also translates into clinical success [18, 19].

## 4. Conclusion

The treatment success depends on adequate medical advice, which takes into account different therapeutic options, autonomy and individuality of each patient, and professional's abilities and available resources. Thus, more studies are needed to analyze the effectiveness of the technique in question, since the inadequate choice of treatment can lead to losses not only for the public and private health systems but mainly for the patient.

## Data Availability

The data used to support the findings of this study are available from the corresponding author upon request.
